# ASPM在肺腺癌中的表达及其与肺腺癌的进展和预后关系的研究

**DOI:** 10.3779/j.issn.1009-3419.2020.01.05

**Published:** 2020-01-20

**Authors:** 俊杰 王, 振宇 何, 仁慧 段

**Affiliations:** 1 453000 新乡，河南省新乡市中心医院肿瘤内三科 Department of Oncology, Xinxiang Central Hospital, Xinxiang 453000, China; 2 510060 广州，中山大学附属肿瘤医院放疗科 Department of Radiotherapy, Cancer Hospital, Sun Yat-Sen University, Guangzhou 510060, China

**Keywords:** 肺腺癌, 人类异常纺锤体样小头畸形相关蛋白, 免疫组织化学, 预后, Lung adenocarcinoma, ASPM, Immunohistochemical, Prognosis

## Abstract

**背景与目的:**

已有的研究表明人类异常纺锤体样小头畸形相关蛋白（abnormal spindle-likemicrocephaly-associated protein, ASPM）是一个与肿瘤发生发展相关的蛋白，ASPM的突变会导致常染色体隐性原发性小头畸形。本研究主要是探讨肺腺癌组织中的表达情况与肺癌进展和预后的关系。

**方法:**

收集90例肺腺癌组织标本和对应的90例肺良性病变组织标本，应用免疫组织化学技术检测其中90对组织中ASPM的表达情况，应用实时荧光定量核酸扩增法和蛋白印迹法的方法检测其中12对组织中ASPM的表达情况。

**结果:**

肺良性病变组织标本中ASPM表达为阴性，肺腺癌组织中ASPM的表达为阳性结果且表达水平均显著高于肺良性病变肺组织（*P* < 0.05）。伴有淋巴结进展的肺腺癌中ASPM的表达水平与不伴有淋巴结进展的肺癌没有差异，无统计学意义（*P* > 0.05）。肿瘤大小≥4 cm的ASPM表达水平均显著高于肿瘤大小 < 4 cm（*P* < 0.05）。分层分析结果表明T分期与ASPM表达层度相关（*P* < 0.05）。肺腺癌组织中ASPM的高表达水平与不良预后呈正相关（*P* < 0.05）。

**结论:**

肺腺癌中ASPM的表达水平明显升高，且与肺腺癌的进展有密切关系。肺腺癌组织中ASPM的表达水平与不良预后呈正相关（*P* < 0.05）。检测肺腺癌中ASPM的表达水平有助于提早的预测肺腺癌的预后。

## Abstract (en)

**Background and objective:**

Previous studies have shown that ASPM is a tumorigenesis associated protein, mutations in ASPM can lead to MCPH. This study mainly explores the relationship between the ASPM expression of lung adenocarcinoma and the development and prognosis of lung cancer. The aim of this study was to investigate the relationship between the expression of abnormal spindle-like microcephaly-associated protein (ASPM) in lung adenocarcinoma and the development and prognosis in lung cancer.

**Methods:**

A total of 90 cases of lung adenocarcinoma tissue specimens and 90 cases of benign pulmonary lesions were collected, the expression of ASPM was detected by immunohistochemical technique, and the expression of ASPM in 12 pairs of tissues was detected by real-time quantity polymerase chain reaction and western blot.

**Results:**

(1) The expression of ASPM in the tissue specimens of benign pulmonary lesions was negative while the expression level of ASPM in lung adenocarcinoma was significantly higher than lung tissue of benign pulmonary lesions (*P* < 0.05). (2) The expression level of ASPM has no remarkable difference in lung adenocarcinoma with lymph node development and the lung cancer without lymph node development, there was no statistically significant (*P* < 0.05). (3) The ASPM expression level in the tumor's size ≥4 cm was significantly higher than than of the size < 4 cm (*P* < 0.05). (4) Hierarchical analysis results show that T stage is related to ASPM expression level (*P* < 0.05). (5) The high expression level of ASPM in lung adenocarcinoma was significantly positively correlated with the poor prognosis (*P* < 0.05).

**Conclusion:**

(1) The expression level of ASPM in lung cancer is obviously increased and closely related to the progress of lung adenocarcinoma. (2) The expression level of ASPM in lung adenocarcinoma was significantly positively correlated with poor prognosis (*P* < 0.05). (3) Detection of the expression level of ASPM in lung adenocarcinoma help to predict the prognosis of lung adenocarcinoma in advance.

肺癌是对人群健康和生命威胁最大的恶性肿瘤之一^[1]^。目前肺腺癌的发病率已经占据所有肺癌病理类型之首。在全球范围内肺癌的发病率和死亡率一直居高不下，国内外有关肺癌预防和治疗的方法也层出不穷。但是早期的诊断和治疗及预后未能显著的提高并解除人类的困扰。一般认为肿瘤发生发展与细胞的增殖迁移等行为相关联^[2]^。ASPM基因是果蝇异常纺锤体的人类直系同源基因，也是最常见的常染色体隐性小头畸形的突变基因。目前的研究发现ASPM在细胞的周期过程中发挥重要作用，文献^[3]^报道，ASPM过表达于恶性肿瘤。本文我们主要是通过文献检索、临床资料的收集及免疫组化等方法。确定ASPM在肺腺癌中的相关性及临床意义。

## 1 材料和方法

### 1.1 研究对象

本研究所用的肺腺癌组织和正常肺组织均来源于河南省新乡市中心医院行手术治疗的患者。本实验研究经过了新乡市中心医院伦理委员会的批准，而且患者本人具有知情同意权。所有的组织都是经手术取下来立刻放入液氮中，并且采用专业组织冻存管快速转移至负八十度冰箱保存。本实验所有的组织均是由固定的两位专业病理科医师证实为肺腺癌，本研究基于患者入院病历收集患者相关情况，统计数据包含患者的一般情况：年龄、性别和患者最终病理类型：肿瘤病理类型、肿瘤病理分级分期、肿瘤位置、肿瘤数目及大小等，并且对患者进行定期随访，关注患者的复发和进展预后情况。

### 1.2 主要试剂

通用性（小鼠/兔聚合物法检测系统）PV-6000二步法检测试剂盒（PV-6000，中杉金桥生物有限公司，北京，中国），浓缩DAB显色试剂盒（ZLI-9018优宁维生物科技股份有限公司上海中国），PBS粉（P80316，百泰克生物科技有限公司，北京，中国），苏木素（G1080，百泰克生物科技有限公司，北京，中国），ASPM兔抗人单克隆抗体（ab238106，Abcam，美国），TRIzol试剂（Thermo, USA），RIPA（R0020，Solaibio，北京，中国），ASPM primer：5′-GGGAAAGGCAAATGGAAAAC-3′，5′-CCCAAGGCCATACAAGTGTT-3′。GAPDH，5′-GAGTCAACGGATTTGGTCGT-3′，5′-TTGATTTTGGAGGGATCTCG-3′（上海生工，中国）等。

### 1.3 免疫组化操作方法及实验主要步骤

首先组织放置于10%的福尔马林固定4 h，之后包埋制作蜡块，蜡块制备完成后放置-20 ℃冰箱1 h左右，然后使用石蜡切片机（LEIZI/515，德国）切片5 μm厚度。切好放置70 ℃烤箱加热约50 min，组织脱蜡二甲苯20 min，无水乙醇10 min，95%乙醇5 min，85%乙醇5 min，75%乙醇5 min，最后切片放置于流水下洗去残留乙醇。PBS洗3遍每次3 min，用枸橼酸钠溶液抗原修复切片高火5 min，中低火8 min-10 min，室温放凉约1 h。PBS再洗3遍每次3 min，3%的内源性过氧化物酶阻断剂每片约40 μL 20 min，PBS再洗3遍每次3 min，在湿盒孵育一抗（1:100），4 ℃，过夜。次日切片恢复室温约30 min，PBS洗3遍每次3 min，室温条件下湿盒内孵育二抗约1 h，PBS洗3遍每次3 min，DAB染色1 min左右，自来水终止染色。苏木素复染约15 s。自来水洗终止染色，脱水、透明：75%乙醇浸泡1 min，85%乙醇1 min，95%乙醇1 min，无水乙醇3 min，二甲苯10 min，最后中性树胶封片，封片后置于通风安全橱晾干约1 h，随后显微镜下观察并采集图片。

### 1.4 结果评定

所有免疫组织化学染色结果由认证专业的2位病理学医师双盲条件下进行评估，并进行图像采集。ASPM的阳性表达定位于细胞质和细胞核，呈淡黄色，黄色或棕黄色。将阳性细胞的百分比和染色效果的强度确定染色结果：遵循随机化原则，以200×放大率捕获10个随机区域或3个代表区域，并计算每个视野中的阳性细胞。百分比取平均值。阳性细胞评定为0分，10%-49%评为1分，50%-74%为2分，≥75%为3分。相同的着色强度也评定为0分-3分，无色评级为0分，灰黄色评级为1分，金黄色评级为2分，棕黄色评级为3分ASPM的ICH评分通过加入强度评分和染色程度评分。通过评分作为观察指数的指标来评估切片上ASPM表达的水平（总分≤3即为低表达，总分 > 3即为高表达）。

### 1.5 实时荧光定量核酸扩增检测（real-time quantity polymerase chain reaction, RT-qPCR）

使用TRIzol试剂（Thermo, USA）按照制造商的说明提组织总mRNA。RNA是反向转录cDNA使用cDNA逆转录试剂盒（Thermo, USA）。采用7900实时荧光定量系统进行mRNA定量PCR（Thermo Scientific, Waltham, MA, USA）。目标基因ASPM primer：5′-GGGAAAGGCAAATGGAAAAC-3′，5′-CCCAAGGCCATACAAGTGTT-3′。GAPDH，5′-GAGTCAACGGATTTGGTCGT-3′，5′-TTGATTTTGGAGGGATCTCG-3′（上海生工，中国）。

### 1.6 蛋白印迹法（Western blot）

使用RIPA（R0020 Solaibio，北京，中国）提取组织中的蛋白质，BCA法测量蛋白浓度，95 ℃ 5 min变性，-20 ℃保存。10% SDS PAGE 90 v电泳约2 h，PVDF膜电转200 mA约2 h，TBST溶液洗3次，每次约10 min，5%脱脂奶粉溶液封闭室温摇床约1 h。TBST溶液洗三次每次约10 min，4 ℃摇床孵育一抗（1:1, 000）过夜。第二天TBST溶液洗3次每次约10 min，孵育二抗（1:1, 000）室温摇床约1 h，TBST溶液洗3次，每次约10 min。ECL曝光并且采集照片。

### 1.7 统计学处理

统计学处理应用SPSS 20.0进行分析，肺腺癌组织及肺部正常组织中的ASPM表达水平和肺腺癌之间不同分期的ASPM表达水平均使用均数±标准差描述，并采用卡方检验分析其表达差异情况。应用*Kaplan-Meier*分析ASPM表达水平与肺腺癌总生存率和无进展生存比率的相关性。以*P* < 0.05为差异具有统计学意义。

## 2 结果

### 2.1 免疫组织化学实验中不同组织间的ASPM表达情况

图 1A中高表达组的组织来源于Ⅲ期-Ⅳ期患者，低表达组的组织来源于Ⅰ期-Ⅱ期患者。免疫组织化学实验结果显示Ⅲ期-Ⅳ期组的ASPM表达水平均显著高于Ⅰ期-Ⅱ期组。并且ASPM是定位于肺腺癌组织中的细胞核并着色表达。图 1B组织是来源于肺部良性病变，免疫组织化学实验结果显示ASPM在肺部良性病变中表达为阴性即不着色。

**1:**
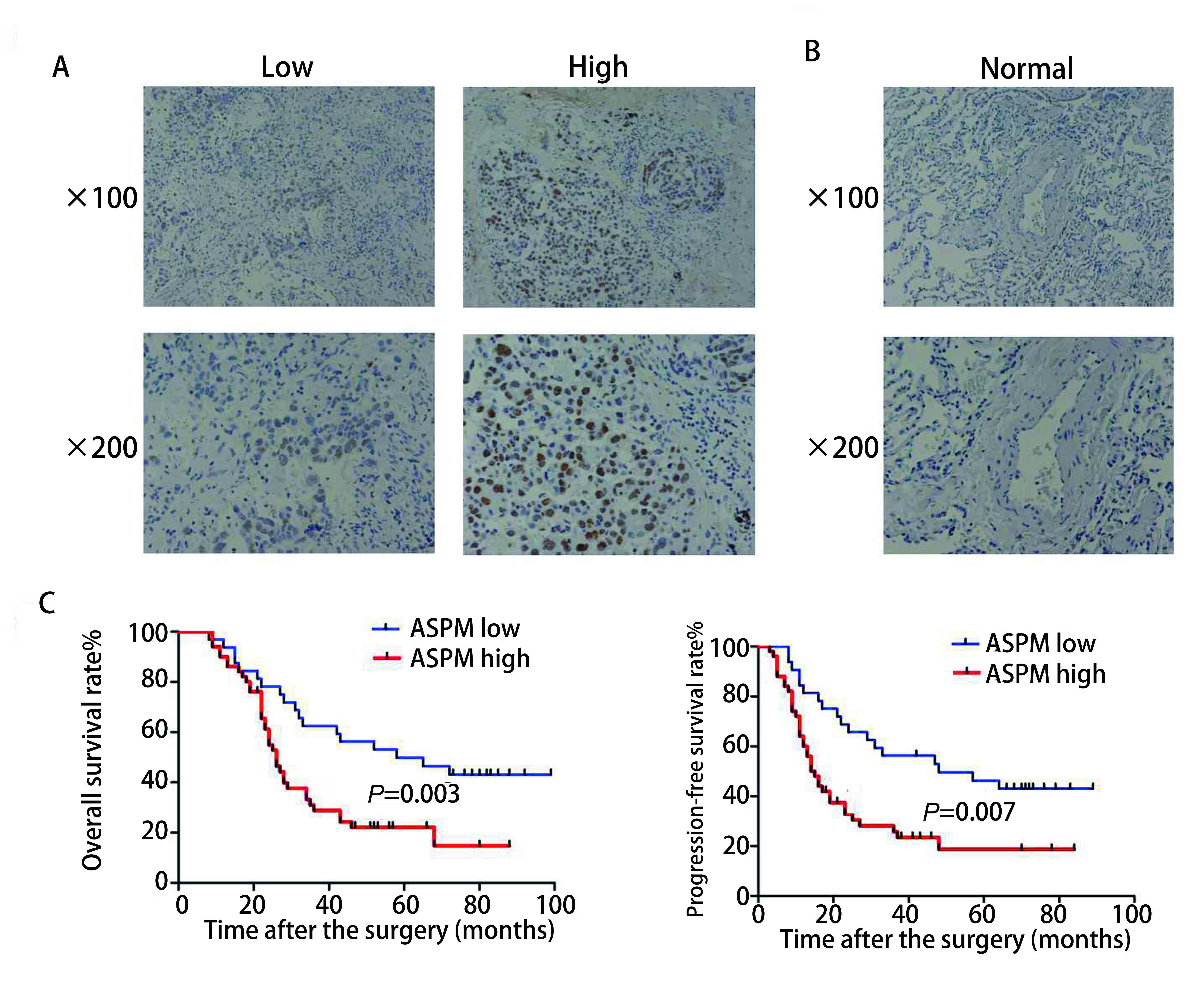
免疫组化检测ASPM在肺腺癌组织（A）和正常肺组织（B）中的表达情况（×100和×200）。C：*Kaplan-Meier*累计总生存率和无进展生存比率曲线分析ASPM高低表达间的差异 Immunohistochemistry to dete c t the e xpression of ASPM in lung adenocarcinoma (A) and normal lung (B) (×100 and ×20 0). C: *Kaplan-Meier* cumulative total survival and progression-free survival ratio curves were used to analyze the difference between high and low expression of ASPM

### 2.2 ASPM在肺腺癌中的表达上调

为了研究ASPM在肺腺癌中的表达，研究小组收集了12对肺腺癌和癌旁组织。我们首先利用RT-qPCR检测上述样本中ASPM的mRNA表达水平（图 2A），发现12对样本中，肺腺癌组织中ASPM的mRNA表达水平高于癌旁组织。随后，我们检测12对组织中ASPM的蛋白表达水平。Western blot结果显示，ASPM蛋白在肿瘤组织中的表达水平高于癌旁组织（图 2B）。结果表明，ASPM在肺腺癌中的转录和翻译表达水平均升高。

**2:**
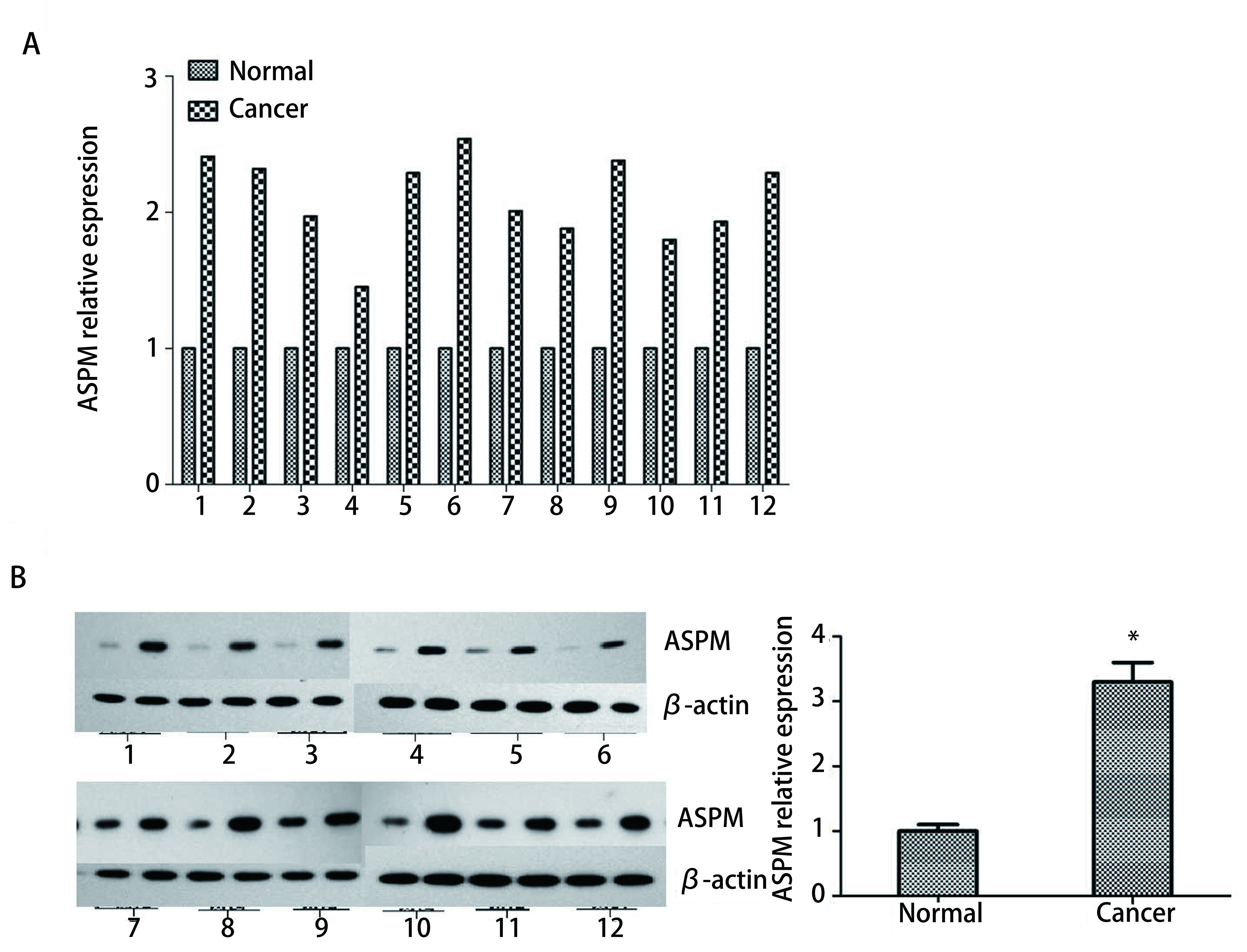
ASPM在肺腺癌组织中上调。A：采用qRT-PCR检测肿瘤患者手术新鲜标本及其邻近组织中ASPM mRNA的表达水平；B：Western blot检测上述标本中ASPM蛋白表达水平。 ASPM is upregulated in lung adenocarcinoma tissues. A: qRT-PCR was used to detect the expression level of ASPM mRNA in surgical specimens of lung adenocarcinoma and its adjacent tissues from tumour patients; B: Western blot was use d to dete c t the protein expression level of ASPM in the abovementioned specimens

### 2.3 分析ASPM表达水平与肺腺癌总生存率和无进展生存比率的相关性

图 1C是对患者术后进行随访时间100个月，并统计分析相关资料。应用*Kaplan-Meier*分析ASPM表达水平与肺腺癌总生存率和无进展生存比率的相关性。结果显示ASPM表达水平在高表达组的肺腺癌患者总生存率远低于低表达组，并ASPM表达水平在高表达组的无进展生存比率显著低于低表达组。

### 2.4 90例肺腺癌患者的临床病理特征与ASPM的关系

表 1基于患者入院病历收集患者相关情况，统计数据包含患者的一般情况：年龄、性别和患者抽烟情况及肿瘤病理分级分期、肿瘤大小淋巴结转移情况等的详细结果。统计结果显示Ⅱ期-Ⅳ期患者的ASPM表达水平均显著高于Ⅰ期-Ⅱ期（*P*=0.021）。肿瘤大小≥4 cm的ASPM表达水平均显著高于肿瘤大小 < 4 cm（*P*=0.024）。ASPM与肺腺癌患者的的病理分期及肿瘤的大小均有显著差异，差异有统计学意义。但是，本研究中患者的抽烟情况、年龄、性别及淋巴结转移与ASPM（*P*值分别为：0.247、0.248、0.478、0.324）的表达，差异无统计学意义。

**1:** 90例肺腺癌患者的临床病理特征与ASPM的关系 Relationships of ASPM and clinicopathological characteristics in 90 patients with lung adenocarcinoma

Clinicopathological characteristics	*N*=90	ASPM expression level		*χ*^2^	*P*
		Low (*n*=36)	High (*n*=54)		
Age (yr)				1.333	0.248
< 55	54	24	30		
≥55	36	12	24		
Gender				0.504	0.478
Male	49	18	31		
Female	41	18	23		
Smoking				1.339	0.247
Yes	66	24	42		
No	24	12	12		
Tumor size (cm)				5.101	0.024*
< 4	40	21	19		
≥4	50	15	35		
Degree of tumor differentiation				1.718	0.190
Low	28	14	14		
High	62	22	40		
Clinical stages				5.316	0.021*
Ⅰ-Ⅱ	28	16	12		
Ⅲ-Ⅳ	62	20	42		
Lymph node metastasis				0.971	0.324
Yes	42	19	23		
No	48	17	31		

## 3 讨论

肿瘤的发生发展是一个多因素、多环节、多阶段的过程。主要包括细胞的异常增殖、侵袭迁移、循环扩散、血管的生成及远处的克隆等。细胞周期的改变是众多因素的关键步骤之一，只有突破对细胞周期的调控才能更有效的抑制肿瘤的发生发展。肺癌不论是在国外还是在中国，不论是在城市还是在农村，肺癌均为最常见的恶性肿瘤之一^[4]^。而且近年来肺癌的发生发展呈不断上升的趋势，而肺腺癌的发生率已经超出其他病理类型的肺癌，比如鳞癌、腺鳞癌、小细胞型肺癌等。目前，人类对于肿瘤的分子生物学基础研究的研究领域主要包括细胞周期调控、miRNA、细胞信号转导等^[5]^。

随着蛋白组学及基因学和一些测序及芯片的发展，生物标志物（biomarker）已经成为近几十年来生命科学研究的热点，大家对于肺癌相关基因及蛋白质有了深刻认识，许多基因及被编码的蛋白（如P21、P27、p53、ras、erbB、hTERT、thymosinp-4、MCM2等）的改变已经被研究发现有利于预测肺癌的预后，并有希望成为肺癌早期诊断的有效方法^[6]^。

*ASPM*基因位于1q31.3上，跨度在62.567 Mb左右，其最长的转录物是由28个外显子组成并编码翻译3, 477个氨基酸大的蛋白质^[7]^。其产物定位于纺锤体极和中心体，包含2个高度保守的N-末端ASNP重复序列、1个氨基酸末端微管结合区、2个同源性钙调蛋白结合区域及1个羧基末端。ASPM主要功能是正确引导又是分裂中纺锤体向两级运动和维持细胞质的均等分裂等。ASPM被认为在有丝分裂纺锤体调节和有丝分裂过程的协调中发挥作用。它被认为与调节神经发生有关。ASPM突变可能导致小头畸形原发性5型（MCPH5）。小头畸形定义为头围比年龄相关的平均值低3个标准差。虽然脑重量和脑皮质体积明显变小，但无明显的神经功能缺损。ASPM的突变会导致常染色体隐性原发性小头畸形（MCPH）^[8, 9]^。有研究^[10-12]^发现ASPM在大脑发育中的神经上皮细胞的均等增殖分裂起着关键因素作用。而ASPM敲低之后NE祖细胞的数量明显减少，这一发现提示MPCH的发生是因为ASPM突变导致有丝分裂调节受损的结果。通过共表达分析，ASPM可能通过调节肿瘤相关的磷酸化作用或者非炎症因子的磷酸化作用而与HCV肝硬化的进展相关，尤其是在癌变过程中。这与我们下一步课题开展提供了新思路。有研究^[13]^发现ASPM在卵巢癌、膀胱癌、肝癌中表达水平上调，和我们免疫组化得出的结果一致，ASPM在肺腺癌中也是表达水平上调而且与肿瘤的发生、侵袭、复发及不良预后相关且呈正相关性。在胶质母细胞瘤、前列腺癌中ASPM的表达水平与肿瘤病理分级临床分期密切相关联^[14]^。这与我们组织化学和临床资料分析出的结果相似，肺腺癌中ASPM的表达水平与肿瘤病理分级临床分期紧密相关。

ASPM可能是一种新的含氧干细胞/祖细胞标志物，可能参与正常胃生理和胃癌的发生。此外，ASPM表达与多发癌临床预后不良有关^[15]^。在肝细胞癌，ASPM表达与血清AFP水平密切相关。尽管ASPM在一些非肿瘤性疾病中发挥重要作用，但尚无研究阐明ASPM在肺腺癌发生与进展中的作用^[16-18]^。目前ASPM在肿瘤中的生物分子机制尚不清楚。在本课题中，我们采用免疫组织化学的实验方法来检测肺腺癌组织与正常肺组织中ASPM的表达水平和临床分期及患者预后的情况。本研究显示癌组织中的ASPM表达水平高于正常组织且随着临床分期的增高，发现ASPM的表达水平也随之增高。最后通过生存分析曲线显示高表达组的ASPM是肺腺癌患者生化复发的危险因素，且与总生存率紧密相关。

但是我们的研究中所纳入的病例数量少的原因可能存在一些因素统计差异并且未进一步研究ASPM在mRNA水平的变化是否与现已经得出的研究结果一致且同样反映肺腺癌的临床分期及不良预后等。与此同时，我们通过查阅文献了解到ASPM和（有爪蟾蜍驱动蛋白2的靶向蛋白）TPX2的表达都与有丝分裂的纺锤体有关。有研究^[19]^发现TPX2在膀胱癌中是激活P53的合成，是通过TPX2-Aurora A复合体调节P53的磷酸化。此外，有研究^[20, 21]^发现在前列腺癌中敲除TPX2能诱导细胞周期静止和凋亡、降低细胞的侵袭能力和抑制细胞的增殖。本研究还发现ASPM与TPX2、BUB1等之间存在极强的相关性，但是它们之间的关系还是空白，因为ASPM在细胞有丝分裂中还可能与某些分子有关联且影响着肿瘤的发生发展，所以我们需要进一步增加纳入病例数量并丰富相关患者临床病理特征资料及实验验证方法深入研究。

综上所述，我们证明了ASPM在肺腺癌中高表达且与临床分期及患者预后相关并对生存期有一定的影响，而ASPM可能是肺腺癌的一项新的独立预后指标。因此，通过检测ASPM的表达可以更好的判断肺腺癌的预后和恶性程度。设想通过抑制ASPM的表达，抑制肿瘤发生发展，改善肺腺癌的不良预后，为肿瘤的靶向治疗提供新的靶点。为肿瘤发病机制研究提供新的思路。

## Author contributions

Wang JJ carried out the experiment of molecular biology and drafted the manuscript. He ZY participated in the design of the study and performed the statistical analysis. Duan RH conceived of the study, and coordination and helped to revise the manuscript.
